# Experimental comparison of platelet-rich plasma with conventional treatment in rats with acoustic trauma induced hearing loss

**DOI:** 10.1016/j.bjorl.2025.101712

**Published:** 2025-09-25

**Authors:** Funda Kutay, Mehmet Ihsan Gülmez, Semsettin Okuyucu

**Affiliations:** Hatay Mustafa Kemal University Tayfur Ata Sökmen Faculty of Medicine, Department of Otorhinolaryngology, Hatay, Turkey

**Keywords:** Acoustic trauma, PRP treatment, Experimantal comparison

## Abstract

**Objective:**

This study aimed to test at an experimental level the contributions of traditional treatments with Platelet Rich Plasma (PRP) to the recovery of hearing in hearing loss due to acoustic trauma.

**Methods:**

Thirty healthy adult rats were used in the study. Otoacoustic Emission (OAE) measurements were performed before the study. All rats with OAE measurements were exposed to 95 dB pure tone sound at a frequency of 4 kHz for 60-minutes. OAEs of the experimental animals were measured again at the 24th hour and it was determined that acoustic trauma had occurred. The rats were divided into 3 groups of 10, the first group was the control group, the 2nd group was determined as the group in which approximately 0.5 mL of intratympanic PRP was injected for 5 doses with 2 days intervals, and the 3rd group was determined as the group in which approximately 0.5 mL of intratympanic steroid was injected with 2 days intervals. OAE measurements made after 7 and 21 days were evaluated, and the treatments given were compared to the group without treatment.

**Results:**

The results obtained from day-by-day and frequency-by-frequency measurements of DPOAE were statistically compared to check whether significant results were obtained. It was determined that better results were obtained in rats with acoustic trauma treated with PRP and prednol compared with acoustic trauma who received no treatment, and close treatment efficacy results were found between PRP and prednol groups.

**Conclusion:**

When DPOAE measurements of these three groups at 4 kHz frequency on various days were compared with Group 1, a significant statistical difference was observed in Groups 2 and 3, but no significant difference was found between Groups 2 and 3. It was concluded that PRP is as effective as steroids in the improvement of hearing.

## Highlights

• The research involving the development of potential therapeutics for acoustic trauma.
• Local experimental studies on rats are guiding our evaluation of PRP efficacy.
• We aimed to make important contributions to the literature and treatment of this disease.

## Introduction

Although damage to the tympanic membrane and middle ear structures resulting from acoustic trauma may resolve spontaneously or be managed surgically, injury to the sensorineural components of the inner ear presents greater complexity and necessitates immediate intervention through various therapeutic strategies. The cochlea, particularly the outer hair cells, constitutes the most vulnerable region of the auditory system in response to noise exposure. Two primary pathophysiological mechanisms ‒ mechanical and metabolic ‒ are implicated. Metabolic injury involves three interrelated processes: The generation of free radicals,^1^ lipid peroxidation,^2^ and cochlear ischemia.^3^ In cases of mechanical trauma, structural disruptions, especially rupture of outer hair cells, are primarily responsible. Noise exposure induces the production of Reactive Oxygen Species (ROS) and Reactive Nitrogen Species (RNS) within hair cells. When the concentrations of ROS and RNS surpass the antioxidative capacity of the cell, oxidative damage to cellular DNA, lipids, and proteins occurs, ultimately leading to apoptosis and permanent sensorineural hearing loss.^4^ Otoacoustic Emissions (OAEs), which are low-intensity acoustic signals originating from the cochlea, can be recorded in the external auditory canal of both humans and animals.^5^ The generation of OAEs is highly dependent on the functional integrity of outer hair cells. Platelet-Rich Plasma (PRP) is an autologous biological product derived from the patient’s own blood and has been utilized in the treatment of various conditions, including nerve injuries, muscle damage, tissue and skin regeneration, and perforations of the nasal septum and tympanic membrane. PRP is obtained by centrifuging blood under appropriate conditions to concentrate platelets and growth factors, and the resulting plasma is injected into the target area to promote and accelerate tissue healing.^6^ PRP serves as an effective delivery system for a variety of growth factors, including Platelet-Derived Growth Factor (PDGF), Transforming Growth Factor-β (TGF-β), Insulin-Like Growth Factor-1 (IGF-1), and Vascular Endothelial Growth Factor (VEGF). Studies have shown that TGF-β2, IGF-1, and VEGF contribute to neural regeneration and upregulation, while PDGF receptors are expressed on neurons. Additionally, PDGF-β has been identified as a mitogen and survival factor for Schwann cells, exerting trophic effects on neurons.^7^

These growth factors facilitate healing by enhancing angiogenesis, and stimulating chemotactic and mitotic activity in tissues, thereby reducing cell apoptosis.^8^

Despite the theoretical advantages and various treatment attempts based on these mechanisms, a definitive and universally effective treatment for noise-induced sensorineural hearing loss has yet to be established, and spontaneous recovery may occasionally occur. Although corticosteroids are widely used as the first-line treatment, their inconsistent efficacy has led to ongoing investigations into alternative therapies. In this context, PRP has gained increasing attention in recent years. Based on this background, the present study aims to evaluate the efficacy of PRP compared to conventional treatments in noise-induced sensorineural hearing loss.

## Methods

### Study authorization

This study was conducted in the Department of Otorhinolaryngology with the approval of the Animal Experiments Local Ethics Committee and the Scientific Research Projects Unit (Ethics Committee Decision No: 109667, Date: 06.12.2021). All experimental procedures involving animals were carried out in accordance with the National Regulations on the Protection and Use of Laboratory Animals.

### Formation of study groups

Thirty Winstar albino adult rats were used in the study. The average weight of the rats was 300‒400 g. The rats were kept in background noise level below 50 decibels (dB) SPL. Ear examinations of all rats were performed under a microscope. The buds in the external auditory canal were cleaned. The external auditory canal and eardrums of all rats used in the thesis were normal in appearance. All rats were kept in a calm environment at an appropriate room temperature (21 °C) and noise level below 50 dB prior to anesthesia. The anesthesia procedure was performed using appropriate gloves and syringe tips to minimize pain. All procedures were performed by the same researcher each day in the early morning hours.

All rats were then anesthetized with intraperitoneal ketamine hydrochloride (40 mg/kg) and xylazine (5 mg/kg).

The rats were divided into 3 groups of 10 rats each and the tails of the rats in all groups were colored with special pens and numbered from 1 to 10. In all groups, one rat died during the 21-day measurements and the results were evaluated over 9 rats. The groups and the procedures applied are shown in Table 1.

**Table 1:** Study groups.

Group	Applied Process	Number
Group 1 (Just noise)	95 dB wideband noise for 4 h	9
Group 2 (Noise + Intra tympanic PRP)	95 dB wideband noise for 4 h + PRP	9
Group 3 (Noise + Intra tympanic prednol)	95 dB wideband noise for 4 h + prednol	9

### Distortion product otoacoustic emission measurements

**Fig. 1:**
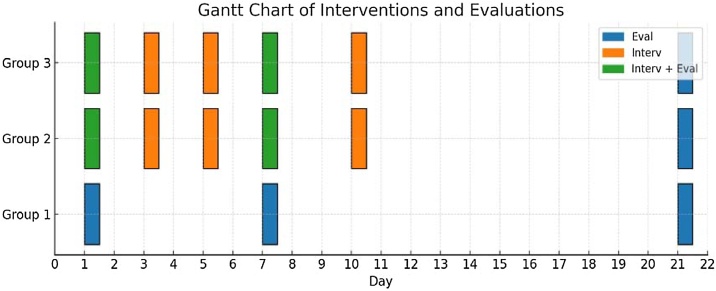
Gantt Chart of Interventions and Evaluations. Blue (Evaluation), Only evaluation; Orange (Intervention), Only intervention; Green (Intervention + Evaluation), Both intervention and evaluation on the same day.

Otoacoustic emission measurement program device was used for distortion product otoacoustic emission measurements. During the recording, the f2/f1 ratio was 1.2 and the intensity level was (L2 = 55 dB, L1 = 65 dB). Signal to Noise Ratio (SNR) at 2f1-f2 frequency was considered positive if it was 6 dB and above. Measurements were performed at 4444 frequencies. DPOAE was measured in all rats. After the first measurements, all groups were exposed to 95 dB SPL broadband noise for 4 h. The noise was applied by means of 2 loudspeakers placed at equal distances on the top of the cabin where the rats were located. After noise exposure, DPOAE measurements of Group 1 rats on the 1st, 7th, and 21st days and DPOAE measurements of Group 2 rats on the 1st, 7th, and 21st days were taken after intratympanic administration of the PRP. PRP obtained by centrifuging the blood taken from the jugular veins of Group 2 rats. We centifruge to first at 160 G for 20 min and then at 400 G for 15 min on the 1st, 3rd, 5th, 7th, and 10th days. In Group 3, similar to Group 2, intratympanic 0.5 mL prednol was administered on days 1, 3, 5, 7 and 10 before post-noise measurements and DPOAE measurements were taken on days 1, 7 and 21. The DPOAE measurements of all groups were taken by the same researcher in an environment where the background noise level was below 50 decibels (dB) SPL, at the same hour. Before starting the experiment, 30 rats with normal otoscopic examination and hearing were randomly selected and divided into three groups. Ear examinations were found to be normal in 30 rats, which were then randomly sampled and divided into 3 groups. 3 groups were followed in separate sections, with each group receiving a drug appropriate for their treatment or being followed as a control group with only DPOAE measurements taken. Intra-group measurements were taken in a blinded manner Fig. 1.

### Obtaining PRP

Platelet-rich plasma was obtained only from the own blood of the rats in Group 2.

**Fig. 2:**
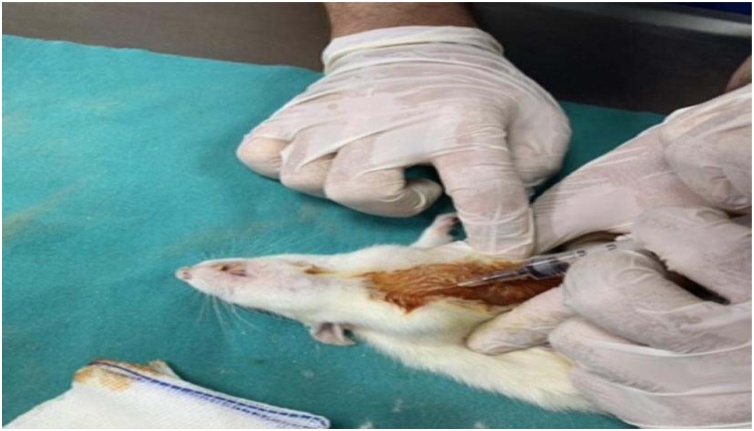
Blood collection from rats.

Firstly, the rats were numbered by painting their tails with special colors. In the presence of a veterinarian, 2 cc of blood was collected from the jugular vein of each rat (Fig. 2).

**Fig. 3:**
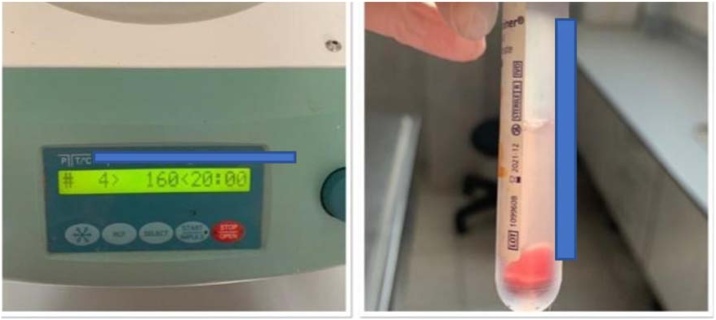
Processing of blood in the centrifuge and the PRP obtained afterwards.

These blood samples were collected in sodium citrate anticoagulant tubes and centrifuged at 160 G for 20 min and then at 400 G for 15 min to separate the PRP-rich portions and stored at +4 °C (Fig. 3).

### Administration of prednol

Intratympanic prednol was administered only to both ears of Group 3 rats. DPOAE measurements were taken after intratympanic administration of approximately 0.5 mL prednol in both ears on the 1st, 3rd, 5th, 7th, 10th days after noise exposure. On day 21, only DPOAE measurements were taken.

### Statistical analysis

IBM SPSS Statistics Version 22 program was used for statistical analysis. Number of units (n), percentage (%), mean ± standard deviation values were used as summary statistics. For independent groups, Man–Whitney *U* was used for mean analysis, chi-square test was used for ratio analysis, and Kruskal–Wallis test was used for the evaluation of all groups. Mc Nemar test was used in the analysis of percentage ratios for dependent groups. Wilcoxon test according to nonparametric distribution was used in the comparison of two means in dependent groups and nonparametric Friedman analysis was used in the analysis of more than two means in dependent groups; p < 0.05 was considered significant. The reduction of one rat in each group did not create a statistically significant difference in the two dependent measurements due to the number of data points being 10 or less.

## Results

DPOAE tests were performed in each group before, immediately after (day 0), 1-, 7- and 21-days after the noise. After the 21st day tests, the rats were sacrificed and their cochleae were removed, but the pathophysiologic results could not be included in the publication because the procedures were interrupted.

### Evaluation of DPOAE results in the group

**Fig. 4:**
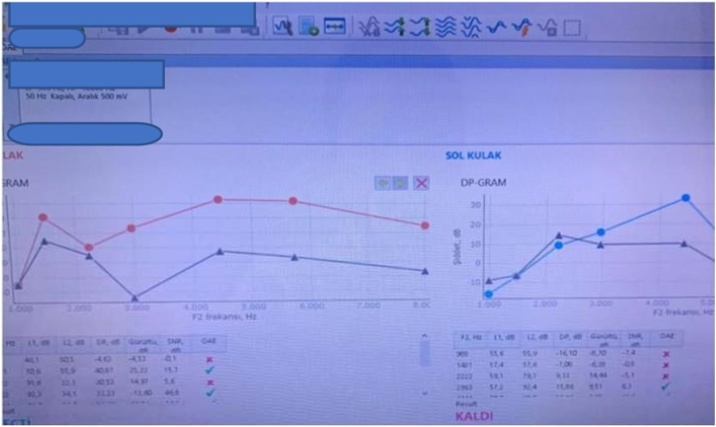
An example of DPOAE applied to a rat in our study is shown.

Otoacoustic emission testing was performed using the DPOAE technique over a wide frequency range Fig. 4. Statistical analysis was performed on the Signal-to-Noise Ratio (SNR) at each frequency separately. Emissions were considered present if the SNR was 6 dB and above. Each test result was evaluated between the groups. In addition to the evaluation of each test result between the groups, the test results were compared with each other within the group and the averages and ratios were measured. Noise-induced hearing loss affects 4 kHz the most and when the hearing frequencies of humans and rats were evaluated; it was determined that the most relevant frequency range was 4–8 kHz.^9^

### Evaluation of DPOAE results between groups

While a significant statistical difference was observed in the bilateral ear DPOAE test measurements of Groups 1 and 2 on days 1 and 7 (p = 0.031, p = 0.003, respectively), no significant statistical difference was observed on day 21 (p > 0.05, Table 2).

**Table 2:** Statistical comparison of hearing gains at 4 kHz for groups 1 and 2.

	Group	Mean Rank	Sum of Ranks	p*
Right 4444 ‒ 0 Day	1	8.94	80.50	0.659
	2	10.06	90.50	
Right 4444 ‒ 1 Day	1	6.78	61.00	**0.031**
	2	12.22	110.00	
Right 4444 ‒ 7 Day	1	5.72	51.50	**0.003**
	2	13.28	119.50	
Right 4444 ‒ 21 Day	1	8.11	73.00	0.269
	2	10.89	98.00	

No significant statistical difference was observed in the bilateral ear DPOAE test measurements of Groups 2 and 3 on days 1, 7, and 21 (p > 0.05, Table 3).

**Table 3:** Statistical comparison of hearing gains at 4 kHz for groups 2 and 3.

	Group	Mean Rank	Sum of Ranks	p*
Right 4444 – 0 Gün	2	8.33	75.00	0.354
	3	10.67	96.00	
Right 4444 – 1 Gün	2	7.06	63.50	0.052
	3	11.94	107.50	
Right 4444 ‒ 7 Gün	2	9.67	87.00	0.895
	3	9.33	84.00	
Right 4444 – 21 Gün	2	9.22	83.00	0.825
	3	9.78	88.00	

While a significant statistical difference was observed in the bilateral ear DPOAE test measurements of Groups 1 and 3 on days 1 and 7 (p = 0.002, p = 0.001, respectively), no significant statistical difference was observed on day 21 (p > 0.05, Table 4).

**Table 4:** Statistical comparison of hearing gains at 4 kHz for groups 1 and 3.

	Group	Mean Rank	Sum of Ranks	p*
Right4444 – 0 Day	1	8.22	74.00	0.309
	3	10.78	97.00	
Right 4444 – 1 Day	1	5.67	51.00	**0.002**
	3	13.33	120.0 0	
Right 4444 – 7 Day	1	5.44	49.00	**0.001**
	3	13.56	122.0 0	
Right 4444 – 21 Day	1	7.78	70.00	0.171
	3	11.22	101.0 0	

**Fig. 5:**
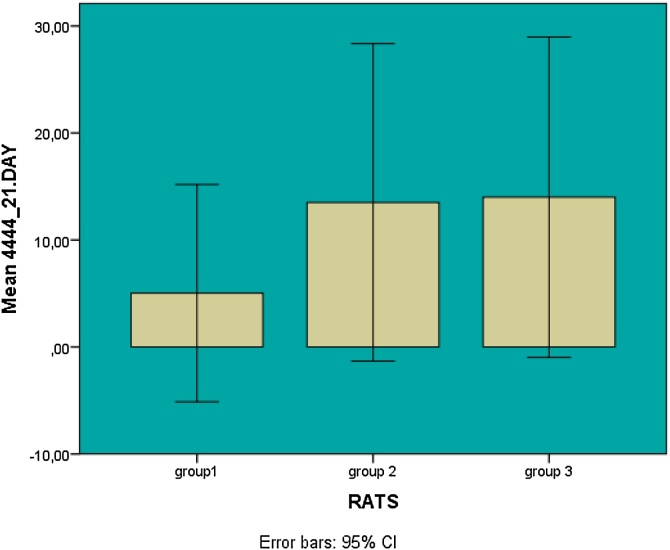
Graphical representation of hearing gains for the 3 groups.

Graphical data including error bars and confidence intervals for the results on day 21 are presented in Fig. 5.

Moreover, p-values across all groups and time points for each frequency were compared and summarized in a Table 5.

**Table 5:** Comparison of hearing gain at certain frequencies for the 3 groups.

	988 frequency	1481 frequency	2222 frequency	4444 frequency	All groups
0 Day	0.303	0.491	0.472	0.499	p*
1 Day	0.956	0.088	0.072	0.003	p*
7 Day	0.028	0.054	0.05	0.001	p*
21 Day	0.041	0.022	0.442	0.344	p*

## Discussion

Research into the development of potential therapeutics for the prevention and treatment of acoustic trauma has been steadily increasing.^10^ However, due to ethical considerations and other constraints, most current studies are conducted using experimental animal models. Animal studies offer a controlled environment, providing valuable insights into both the short- and long-term effects of acoustic trauma on the auditory system. These models form the basis for much of the knowledge currently applied in therapeutic interventions.

Rats are commonly used in auditory research due to their physiological similarities to the human auditory system, as well as their availability and low cost. Among these, Wistar albino rats, which were first developed at the Wistar Institute in 1906 for biological and medical research, are one of the most frequently utilized strains in noise-induced hearing loss studies.^11^

However, experimental conditions in this field vary widely. Variables such as the intensity and duration of noise exposure, the pharmacological agents administered, the timing of interventions, and the auditory parameters measured can differ significantly across studies. It has been demonstrated that noise levels of 85 dB SPL and above are sufficient to cause acoustic trauma.^12^ The duration of noise exposure used to induce trauma in experimental models is also variable, ranging from 30 min to several hours, and in some cases, involving repeated exposures over multiple days.^13^^,^^14^ For instance, Cascella et al. applied 6 kHz white noise at 115 ± 3 dB SPL for 2 h to evaluate the therapeutic potential of Acuval 400 in acoustic trauma.^15^ Similarly, Aksoy et al. examined the effects of thymoquinone in a rat model by exposing animals to 105 dB SPL white noise for 4 h.

Studies have shown that narrowband and pure tone noises, where the energy is concentrated within a single octave band, tend to cause more localized cochlear damage, whereas broadband noise (white noise), which distributes energy evenly across all frequencies, results in uniform damage throughout the cochlea.^16^ Despite this, neither noise intensity nor exposure duration has been fully standardized across studies.

In our study, we exposed all experimental groups to 95 dB SPL broad band (white) noise for 4 h.

Otoacoustic Emissions (OAEs) are low-level acoustic signals generated by the outer hair cells of the cochlea and can be measured from the external auditory canal via a sensitive microphone placed within a probe. Among the various OAE tests, we specifically employed Distortion Product Otoacoustic Emissions (DPOAE) due to its sensitivity and specificity. In DPOAE testing, two distinct pure-tone frequencies are presented simultaneously, resulting in intermodulation distortion within the cochlea. These distortion products are then recorded from the external auditory canal and provide functional information about the integrity of the outer hair cells.^17^^,^^18^ Importantly, DPOAE responses observed in rodent models, such as rats, have been shown to closely resemble those seen in humans, further supporting the translational relevance of this model.^19^

When the hearing frequencies of humans and rats were evaluated, it was found that the most relevant frequency range was 4–8 kHz.^9^ Although there is no standardization in the creation of hearing loss at the experimental level, measurements were generally taken on the 1st, 7th and 21st days of the measurement periods and the changes in hearing were examined. After the experimental occurrence of acoustic trauma has been determined by tests such as DPOAE, various medical treatments have been tested on rats, their treatment efficacy has been examined and different treatment methods have been tried to be developed. Zhou et al. compared inratympanic saline with glucocorticoid administration after acoustic trauma in animals and observed that hearing improved statistically in those treated with glucocorticoid.^20^ Some studies have shown that local PRP has a nerve protective effect. Chou et al. showed that PRP applied to animals was effective in facial nerve regeneration.^21^ In another study, regeneration was detected in the nerve as a result of PRP application to cavernous nerve defect developing in rats.^22^ Basic principle of PRP therapy is to inject concentrated platelets at the site of injury to initiate tissue repair by releasing a variety of bioactive factors (growth factors, cytokines, lysosomes) and adhesion proteins, which are responsible for initiating the hemostatic cascade reaction, synthesis of new connective tissues, and revascularization.^23^ Released growth factors bind to the outer surface of the cell membrane of the target cell via transmembrane receptors on the cell membrane. These transmembrane receptors in turn induce the activation of endogenous signaling proteins that further activate intracellular second messengers. The latter induce various intracellular gene expressions such as cell proliferation, matrix formation and synthesis of collagen.^24^ This mechanism is considered to play a particularly important role in nerve regeneration. Although there are rat studies that have measured hearing improvements with local intratympanic injections similar to our study, a one-to-one experimental comparison of the newly developed PRP and prednisol used in routine treatment has not been observed in our literature review. We anticipate that our study will be very unique and pioneering in this context. We think that it will make important contributions to future animal and human studies. In recent studies, there is a certain standardization in obtaining PRP from rats. Extraction of PRP from rats has become standardized with the protocol developed by Sonnleitner et al.^25^ We have obtained PRP by following this protocol in our thesis. Approximately 2 cc of blood obtained from the jugular vein of rats was firstly centrifuged at 160 G for 20 min and then the platelet rich concentrate was taken into a separate tube and centrifuged at 400 G for 15 min. In addition, since blood was taken from the jugular vein, the rats were not harmed. The material obtained was applied directly on the 1st day and on the 3rd, 5th, 7th, 10th and 21st days, each rat's own PRP, which was kept in the refrigerator at +4 degrees, was activated with calcium chloride and applied to the same rat again. In our study, OAE results were compared within and between groups, and a statistically significant difference was observed between days only in Group 2, where PRP was applied. No significant difference was observed in the other two groups. In statistical comparisons between groups, a significant statistical difference was observed between Group 1 and the other two groups, but no significant difference was observed between Groups 2 and 3. Studies directly comparing the histopathological mechanisms of action of steroids and Platelet-Rich Plasma (PRP) are scarce in the literature. In a rabbit tendinitis model, Zamirbekova et al.^26^ demonstrated that both PRP and steroids exhibited comparable short-term effects on inflammatory response, cellular proliferation, and tissue regeneration. However, in the long term, steroid treatment appeared to provide greater histopathological improvement in these processes. To date, no study has been found that directly compares these two groups in terms of auditory nerve regeneration. Increasing the number of such studies would contribute significantly to a better understanding of their underlying mechanisms of action.

It has been suggested that in sensorineural hearing loss, there is an increase in cellular degeneration particularly in the organ of Corti, hair cells, and the stria vascularis.^27^ In experimental studies involving rats with sensorineural hearing loss treated with PRP, appropriate cochlear excision and subsequent pathological examination would allow for cellular-level assessments of these structures, thereby providing valuable insights into the therapeutic effects of the treatment. Additionally, increasing the sample size would contribute to enhancing the statistical reliability of the findings.

In our study, an evaluation was made to compare the effectiveness of our treatments given to Wistar rats, which were followed up under appropriate conditions, evaluated in terms of hearing and found to be healthy, with frequent DPOAE measurements taken after experimental acoustic trauma. The results showed a significant improvement in the percentage ratio in the treated PRP and prednol groups compared to the control group (p < 0.031 and 0.003 ‒ p < 0.002 and 0.001). Similar treatment efficacy results were obtained between the PRP and prednisol groups (p > 0.05, meaning that the newly trialed prp was highly as effective as prednisol used in standard treatment.

## Conclusion

When DPOAE measurements of these three groups at 4 kHz frequency on various days were compared with Group 1, a significant statistical difference was observed in Groups 2 and 3, but no significant difference was found between Groups 2 and 3. It was concluded that Prp may offer comparable efficacy to steroids. The absence of a statistically significant difference on day-21 may be attributed to the limited sample size or to the onset of a spontaneous recovery phase in the rats. To improve the reliability of the findings, it would be advisable to either repeat the exposure frequency using the non-standardized hearing loss model or to conduct a preliminary pilot study in which the rats are monitored for 21 days, thereby allowing for the selection of a model that induces sustained hearing loss. Furthermore, excising the cochleae for histopathological examination would provide additional insights and contribute to the robustness of the results. Nevertheless, conducting more extensive studies with a larger sample size to evaluate this result would be much more beneficial in supporting the findings and increasing their reliability.

## ORCID ID

Funda Kutay: 0000-0001-6257-9183

Semsettin Okuyucu: 0000-0001-8552-2403

## Declaration of competing interest

The authors declare no conflicts of interest.
